# Nutrition in Gilbert’s Syndrome—A Systematic Review of Clinical Trials According to the PRISMA Statement

**DOI:** 10.3390/nu16142247

**Published:** 2024-07-12

**Authors:** Zuzanna Goluch, Aldona Wierzbicka-Rucińska, Ewelina Książek

**Affiliations:** 1 Department of Food Technology and Nutrition, Wroclaw University of Economics and Business, Komandorska 118/120, 53-345 Wrocław, Poland; 2 Department of Clinical Biochemistry, Radioimmunology and Experimental Medicine, Children’s Memorial Health Institute, 04-730 Warsaw, Poland; 3 Department of Agricultural Engineering and Quality Analysis, Wrocław University of Economics and Business, Komandorska 118/120, 53-345 Wrocław, Poland; ewelina.ksiazek@ue.wroc.pl

**Keywords:** Gilbert syndrome, Gilbert disease, diet, nutrition, caloric restrictions, fasting, PRISMA

## Abstract

Gilbert syndrome is the most common hyperbilirubinemia, associated with a mutation in the UGT1A1 bilirubin gene, which produces an enzyme that conjugates bilirubin with glucuronic acid. Episodes of jaundice occurring in GS negatively affect patients’ quality of life. This systematic review aimed to analyze clinical studies regarding nutrition in people with GS. The study followed the PRISMA guidelines and utilized the Ebsco, Embase, Cochrane, PubMed, Scopus, and Web of Science databases to search clinical trials focused on diet/nutrition in GS (1963–2023 years). The methodological quality of selected studies was assessed using the Jadad scale. As a result, 19 studies met the inclusion criteria. The research mainly focused on the impact of caloric restriction, consumption of various diet variants, and vegetables and fruits on hyperbilirubinemia and metabolic health. A nutritional intervention consisting of not applying excessive calorie restrictions and consuming fats and biologically active compounds in vegetables and fruits (*Cruciferae*, *Apiaceous*, *Rutaceae*) may prevent the occurrence of jaundice episodes. It is justified to conduct further research on detecting such compounds in food, which, by influencing the expression of the UGT liver enzyme gene, could contribute to regulating bilirubin concentration in the blood of people with GS.

## 1. Introduction

Gilbert and Lereboullet first described the syndrome in 1901 as “La cholemie simple familiale”. Meulengracht “rediscovered” the syndrome in 1939 in a published paper titled “Icterus intermittens juvenilis” [1,2]. Gilbert’s syndrome (GS) is an example of a genetically induced, nutritionally exacerbated metabolic disorder occurring without liver cell damage or hemolysis. GS has long been described as an asymptomatic disorder with no clinical implications. However, studies have shown that jaundice episodes occurring in GS affect patients’ quality of life. Kamal et al. [3] demonstrated significant differences in vitality, emotional role, social functioning, worries, and general health status during jaundice episodes in individuals with GS, as measured by the SF-36v2 (Short form-36 Health Survey test, version 2) and CLDQ (Chronic Liver Disease Questionnaire) tests, compared to a control group.

GS is a genetically determined disorder. Because its course can be asymptomatic, the disease may remain undiagnosed until the second decade of life, when a patient may develop mild hyperbilirubinemia with jaundice features [4]. Hyperbilirubinemia typically manifests during puberty, although it is believed that this condition causes some severe jaundice episodes in the neonatal period.

GS is the most commonly described hyperbilirubinemia, a benign disease that occurs worldwide, though its frequency varies depending on population and ethnic background. The prevalence of GS is estimated to be 3–16% in the general population [1,3,5], 2% in East Asian populations, and 20% in India, South Asia, and the Middle East. Considering ethnic groups, the prevalence of GS in Caucasians is estimated at 2–10% [5]. GS is diagnosed more frequently in men than in women (4:1), with a protective role attributed to hormones-estrogens. Radu and Atsmon [1] also estimated that GS occurs more frequently in men (~12.4%) than in women (~4.8%), which is explained by increased production of unconjugated bilirubin during puberty due to changes in the synthesis of steroid sex hormones (androgens) that inhibit enzymatic glucuronidation [6].

The pathogenesis of GS is linked to the production of bilirubin, a byproduct of hemoglobin catabolism, which is excreted with bile into the gastrointestinal tract. The “bottleneck” in bilirubin elimination is its hepatic conjugation with glucuronic acid, mediated by the enzyme UGT1A1. This reaction converts bilirubin into a bile-soluble compound rapidly excreted into the intestine [7]. GS is likely inherited in an autosomal dominant manner with incomplete penetrance. The pathogenesis of GS is determined by a mutation in the UGT1A1 gene, which encodes UDP-glucuronosyltransferase, reducing its activity by up to 80%. This enzyme is crucial in converting free bilirubin into a form conjugated with glucuronic acid [2,8,9]. A defect in UDP-glucuronosyltransferase hampers the uptake of unconjugated bilirubin at the vascular pole of hepatocytes and its conjugation with uridine diphosphate glucuronic acid. The reduced activity of this enzyme results in decreased conjugation, leading to an increase in the total bilirubin concentration in the blood serum, which prevents its excretion in bile. The mutation in the promoter region of the UGT1A1 gene involves an increased number of thymine–adenine repeats. Most patients (approximately 90%) with GS have a homozygous A(TA)7TAA genotype, meaning a seven-repeat (TA) sequence in both alleles of the UGT1A1 gene. Common polymorphic mutations in GS include a TA insertion mutation in the TATA box [A(TA)7TAA] (UGT1A1*28) and c.211 G>A (p.G71R) in exon 1 (UGT1A1*6). In Caucasian and African populations, almost all GS individuals are homozygous for UGT1A1*28. In Japanese, Chinese, and Korean populations, an additional mutation, UGT1A1*6, exists, which causes GS in the homozygous state [5]. The prevalence of this mutation in the Caucasian population is approximately 16%. The homozygous A(TA)7TAA polymorphism is more frequently reported in Caucasian populations than in Asian populations [10]. However, it is estimated that about 10% of individuals with GS have a heterozygous 6/7 genotype, meaning they possess six TA repeats in one allele and seven TA repeats in the other. These individuals likely have additional mutations in the UGT1A1 gene or other genes that contribute to the manifestation of the disease. Healthy individuals typically have the A(TA)6TAA genotype with six TA repeats, although alleles with five or eight TA repeats are extremely rare [3]. Caucasian wild-type homozygotes UGT1A11 (6/6) or heterozygotes 6/7 have about half the total serum bilirubin concentration compared to homozygotes 7/7 (UGT1A1*28), as confirmed in vivo through enzyme studies on human liver tissue [11]. The formation of bilirubin glucuronide by UGT1A1 was 80% and 50% for genotypes 6/7 and 7/7, respectively, compared to the 6/6 genotype.

The factors most commonly triggering hyperbilirubinemia include being dehydrated, fasting, not getting enough sleep, being stressed, having surgery, hemolytic reactions, febrile illnesses, menstruation, and overexertion. Reducing daily caloric intake by 400 calories can lead to a 2–3 fold increase in blood bilirubin levels within 48 h. A similar increase in bilirubin levels can occur with a normocaloric diet lacking lipid supplementation. However, with a normal diet, bilirubin levels usually return to physiological norms within 12 to 24 h [2]. In the 1990s, it was demonstrated [12,13] that fasting during the perioperative period and the use of pain relievers (acetaminophen) post-surgery exacerbated bilirubinemia in patients with GS. However, a case study [14] suggested that the use of general anesthesia (halothane, thiopental, and ketamine) for surgery worsened bilirubinemia. In contrast, inhalational anesthesia (isoflurane) or general intravenous anesthesia with propofol and remifentanil did not impair liver function. More recent studies by Kamal et al. [3] indicated that risk factors for the occurrence of clinical jaundice in adult Egyptians with GS included general anesthesia, pregnancy (elevated levels of unconjugated bilirubin were detected between the 10th and 16th weeks of pregnancy and remained high throughout pregnancy and part of the postpartum period), fasting for more than 12 h, low-calorie weight loss plans (<1200 kcal), systemic infections, and intense physical exertion (such as soccer, tennis, fast swimming, or weightlifting).

GS typically does not cause significant clinical symptoms, aside from episodic hyperbilirubinemia predominantly featuring unconjugated bilirubin (UCB), which usually occurs after exertion or overeating. The first symptom is generally the periodic yellowing of the skin and sclera without skin itching. However, in some patients, the severity of hyperbilirubinemia can be associated with malaise, weakness, loss of appetite, abdominal discomfort, indigestion, flu-like symptoms, nausea, vomiting, concentration difficulties, sweating, anxiety, or palpitations [3,15].

In diagnostic terms, Gilbert’s syndrome (GS) may be identified by a mildly elevated total bilirubin (TB) concentration in serum, with normal levels of liver transaminases (ALT, AST, GGTP, ALP), markers of bile duct function, and red blood cell count [5]. A characteristic feature of GS is unconjugated hyperbilirubinemia, typically not exceeding 6 mg/dL (100 μmol/L). Bilirubin concentrations below 5 mg/dL, predominantly consisting of unconjugated bilirubin (“indirect bilirubin”) or with conjugated bilirubin (“direct bilirubin”) < 0.7 mg/dL (excluding hemolysis), are indicative of GS [16]. The diagnosis of GS is based on repeated measurements of total and indirect bilirubin over 12–24 months. In patients with GS, a characteristic configuration is observed: a persistent increase in total bilirubin concentration with a predominance of the indirect fraction alongside average concentrations of aminotransferases (ALT, AST), GGTP, ALP, and bile acids. Bilirubin levels in individuals with GS often fluctuate and increase under excessive physical exertion and stress (infectious diseases, dietary indiscretions, exam periods, etc.).

Diagnosed GS does not require treatment. Occasionally, attempts have been made to use phenobarbital to induce UDP-glucuronosyltransferase activity and reduce bilirubin levels in the blood; however, the effectiveness of this approach is not always satisfactory. Generally, the prognosis for GS is good as the condition does not lead to liver damage or systemic consequences. Nevertheless, reports indicate that individuals with impaired UDP-glucuronosyltransferase activity may experience exacerbated adverse reactions when using certain medications such as nonsteroidal anti-inflammatory drugs (NSAIDs), antivirals, and anticancer drugs. Therefore, dosages of certain medications, such as paracetamol and ibuprofen, should be individually adjusted in individuals with GS, as standard doses may induce toxic effects. Additionally, adults with GS are significantly more likely to have concurrent schizophrenia compared to the general population. About 50% of GS patients have concurrent hemolytic anemia, increasing their risk of developing gallstones and bile duct stones [17]. Kamal et al. [3] demonstrated among Egyptian adults that the frequency of food intolerance disorders, such as celiac disease and lactose intolerance, was significantly higher in individuals with GS compared to healthy individuals. In contrast, the prevalence of other coexisting conditions (diabetes, rheumatoid arthritis, and hypertension) was similar to the general population.

Therefore, the primary aim of this paper is to provide a systematic review of clinical research on nutrition/diet in Gilbert’s disease. Through rigorous selection of relevant data, this study shows the changes that have occurred over 60 years in the approach to nutrition and the resulting health benefits.

## 2. Materials and Methods

This review was reported following the Preferred Reporting Items for Systematic Reviews and Meta-Analysis (PRISMA) [18].

The study for this systematic review was restricted to clinical trials carried out in humans in the past 60 years (1964–2023). Six databases were selected for literature research: Ebsco, Embase, Cochrane, Pub Med, Scopus, and Web of Science. The search and selection process was performed by three reviewers working independently and in parallel.

Searches included a combination of MeSH terms and keywords, applying quotes and field tags with BOOLEAN operators. Not all databases had the same query options (for example, selecting a clinical trial), which is why stages and phrases were created. For all databases, five primally exclusion steps were determined: (1) keywords (“Gilbert* Syndrome” OR “Gilbert* Disease” AND diet* OR nutrit* AND clinic*); (2) year (1964–2024); (3) publication type (article); (4) language (English); (5) source type (journal).

**Figure 1:**
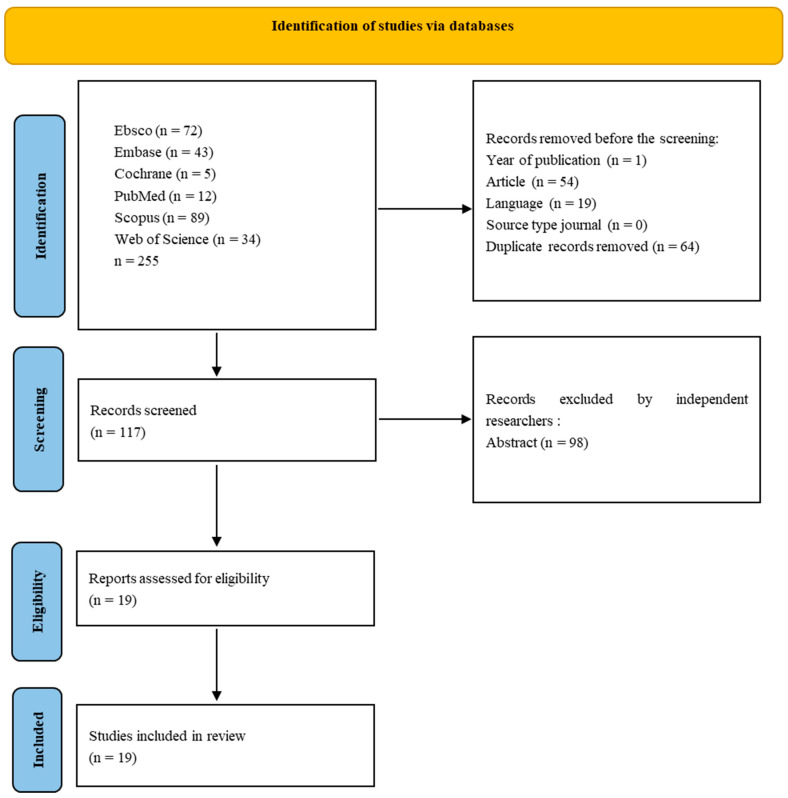
PRISMA flow diagram representing the screening strategy and selection process for research articles. Process of selecting eligible studies.

For non-medical-only databases, screening was conducted in the following areas: Title, Abstract, Keywords (Scopus–Core Collection), Topic (Web of Science–Medline), and Ebsco (All). The last search was conducted in February 2024 because some publishers delayed publishing articles from 2023. Results from each database were exported to files with the appropriate extension of CSV or Excel, and summaries were created for each database containing publication information, including abstracts. The results files are included in the Repository for Open Data (https://doi.org/10.18150/RA5ILC). The initial search resulted in 255 studies. After removing non-English publications and reviews and deleting duplicates, 117 records were selected for abstract review. Among these, 120 studies were excluded according to the exclusion criteria, and the full text of 19 eligible studies was included—only studies involving humans. Articles concerning genetic, pharmacological, or simulation research were excluded from the review. Three independent researchers reviewed the abstracts, and papers that did not meet the criteria for inclusion in the review were excluded through discussion. The literature search process is presented in Figure 1.

The Jadad scale was used to assess the quality and reliability of study outcomes in selected clinical trials [19,20,21]. This scale encompassed three main categories: randomization (0 points for quasi RCTs; 1 point if randomization was declared but without detailed description of random number generation; 2 points if correct random number generation method was described), double blinding (0 points if blinding was not mentioned; 1 point if blinding was mentioned but effectiveness was not described; 2 points if the study employed a robust method such as placebo to ensure double blinding), and withdrawals and dropouts (0 points, if withdrawals were not described despite fewer, analyzed patients compared to recruited ones). Evaluation using the Jadad scale categorized scores of 0–2 as indicative of low-quality clinical trials, 3 as medium-quality, and 4–5 as high-quality clinical trials.

## 3. Results

### 3.1. Caloric Restriction in Gilbert’s Syndrome

Among the selected publications, 12 focused on caloric restriction (Table 1). These studies were conducted with healthy participants as well as individuals with Gilbert’s syndrome, liver diseases, and hemolytic anemia. Participants were divided into groups based on age, gender, and ethnicity. A basic diet (2000–3000 kcal), a restrictive diet (≤400 kcal, with varying macronutrient compositions) supplemented or not with nicotinic acid, and a refeeding diet (2300 kcal) were administered. The duration of the restrictive diets and observation periods ranged from 11 h to 1 year.

In the 1970s, Felsher et al. [22] conducted a study involving seven adult patients with Gilbert’s syndrome (GS), in which they implemented three dietary phases: baseline, caloric restriction, and refeeding while monitoring changes in serum bilirubin levels. The baseline diet ranged from 2000 to 3000 calories and lasted 48 to 72 h. Subsequently, calorie intake was reduced to fewer than 400 calories (either as a low-calorie carbonated beverage or skim milk or orange juice) for 36 to 72 h (extended to 7 days in 2 studies). After the caloric restriction phase, the diet rapidly increased to baseline levels (refeeding). The study also included six relatives of GS patients and eight healthy individuals as controls. The mean serum bilirubin levels in patients significantly (*p* < 0.01) differed before caloric restriction, during restriction, and refeeding periods (1.7 vs. 4.7 vs. 2.1 mg/100 mL, respectively). A correlation between calorie intake and serum bilirubin levels was observed. A rapid increase in serum unconjugated bilirubin levels occurred within 24 h of initiating short-term fasting. Bilirubin levels markedly decreased within 12 to 48 h after resuming high-calorie intake. Among the eight healthy control subjects, fasting resulted in only a slight increase in serum bilirubin levels. In three out of six relatives of a GS patient, a 48 h fast led to a significant rise in serum bilirubin levels, although they did not have jaundice. The authors of the cited studies demonstrated an association between calorie intake, hyperbilirubinemia, and fluctuations in serum bilirubin levels in GS.

In subsequent studies in the 1970s, Owens and Sherlock [23] aimed to determine whether reducing calorie intake and its effect on blood bilirubin levels could serve as a simple diagnostic test for diagnosing GS compared to healthy individuals or those with other liver diseases and hemolytic anemia. The authors hypothesized that increased blood levels of unconjugated bilirubin (UCB) must be caused by either increased production during hemolysis, decreased hepatic clearance, or a combination of both factors. They conducted a study involving 37 individuals divided into four groups: healthy patients, patients with GS, those with liver diseases, and those with hemolytic anemia. Before starting the diet, they measured the blood’s total bilirubin (TB), hemoglobin (HGB), and cortisol levels. Patients received a basic diet for the first two days, then a diet reduced to 400 calories/day for the next three days. Blood samples were taken daily to measure TB, UCB, conjugated bilirubin (CB), haptoglobin, and cortisol levels. Patients were administered indocyanine green intravenously (0.5 mg/kg body weight), and its plasma disappearance was measured after 8 h and a 36 h fast in four healthy individuals and one patient with GS. Cortisol levels were measured in plasma before the diet and 72 h after the low-calorie diet was administered. Additionally, a study was conducted on Sprague-Dawley rats: the first group (n = 7) received a basal diet for three days, while the second group (n = 7) was starved. It was found that the average plasma TB concentration in rats on a normal diet was 0.5 mg/100 mL. In comparison, reduced calorie intake increased bilirubin to 0.8 mg/100 mL after 48 h of calorie reduction but did not exceed 1.0 mg/100 mL. A significant (*p* < 0.001) increase in UCB concentration from 0.3 to 0.5 mg/100 mL was observed, but there were no changes in CB levels. TB concentration significantly increased in GS patients with elevated UCB levels in plasma (from 1.8 to 3.5 mg/100 mL) within 24 h of calorie restriction. UCB levels significantly rose in blood after 48 h of starvation (from 1.5 to 3.4 mg/100 mL), with some individuals experiencing an increase of up to 100% compared to basal diet conditions. There were no significant changes in CB concentration in the blood. The average increase in plasma bilirubin concentration after implementing dietary calorie reduction was significantly higher in GS patients (110%) compared to healthy individuals (60%). Meanwhile, TB concentration in plasma averaged 2.3 mg/100 mL in patients with liver diseases under basal diet conditions, and UCB levels were similar to those in GS patients (1.3 mg/100 mL). However, reducing calorie intake in the diet did not significantly decrease TB and UCB concentrations in plasma. Similarly, no significant changes in bilirubin levels were observed in patients with hemolytic anemia after calorie reduction in the diet. None of the patient groups showed significant changes in haptoglobin and cortisol concentrations after implementing the calorie-restricted diet. There were also no significant changes in indocyanine green disappearance time in half of the patients after 36 h of fasting following the marker injection. Moreover, studies on rats have demonstrated that hepatic bilirubin uridine diphosphate glucuronosyltransferases activity (per gram of liver; per gram of nitrogen) was significantly (*p* < 0.01) lower in starved rats compared to those allowed food. According to the authors, individuals with GS can be distinguished from healthy individuals or patients with liver diseases or hemolytic anemia by implementing a 400-calorie reduction in the diet, as they exhibit further significant increases in UCB in serum, but not when they have normal TB levels. The lack of changes in haptoglobin concentration after the reduced-calorie diet excluded the hypothesis of hemolysis as the cause of hyperbilirubinemia. Similarly, the absence of significant changes in indocyanine green clearance did not indicate reduced liver blood flow and reduced hepatic clearance of bilirubin. Starvation in rats led to a significant impairment of uridine diphosphate glucuronosyltransferase activity in the liver, not due to generalized liver protein catabolism. This activity is lower in individuals with GS.

Bensinger et al. [24], inspired by Gilbert’s 1906 hypothesis regarding increased heme turnover during caloric deprivation in individuals with Gilbert’s Syndrome (GS), investigated its impact on endogenous carbon monoxide (CO) production. They posited that CO and bilirubin are produced equimolarly during heme catabolism, suggesting that measuring CO levels could reflect this process. The study included 14 participants divided into two groups: a control group (n = 5, both sexes) and a GS group (n = 9, only men). Both groups underwent a 48 h 400-calorie diet containing approximately 46 g carbohydrates, 16.5 g protein, and 14.5 g fat. Blood samples were collected before and after the diet to measure serum total bilirubin (TB) concentrations and endogenous CO production rates. Significant findings included a notable (*p* < 0.07) 77% increase in TB levels in the control group after caloric restriction (0.44–0.78 mg/100 mL). At the same time, CO production showed no significant change (from 0.34 to 0.45 µmol/h/kg BW). In contrast, the GS group exhibited a significant (*p* < 0.001) 138% increase in serum TB levels (from 1.3 to 3.1 mg/100 mL), with no statistically significant change in CO production (from 0.41 to 0.47 µmol/h/kg BW). These results led the authors to conclude that the rise in serum bilirubin levels following caloric restriction does not stem from increased heme turnover.

Felsher and Carpio [25] conducted a dietary study involving 10 patients with Gilbert’s Syndrome (GS), 11 patients with hemolysis and unconjugated hyperbilirubinemia, and 13 normal subjects. Participants underwent a 2-to-3-day baseline diet providing 2500 to 3000 cal/day, followed by a sudden reduction in caloric intake to approximately 300 cal/day (consisting of approximately 30 g carbohydrates, 20 g protein, and 10 g fat) for two days (fasting). Blood samples were collected daily before breakfast to measure serum total bilirubin (TB) concentrations (prefasting level). Significant (*p* < 0.01) increases in serum TB levels were observed in both GS patients and normal subjects following caloric restriction, with a significantly (*p* < 0.001) greater increase noted in GS patients (from 1.5 to 3.5 mg/100 mL) compared to normal subjects (from 0.5 to 0.9 mg/100 mL). Patients with hemolysis also showed an increase in serum TB levels, averaging from 2.8 to 3.5 mg/100 mL, which was significantly (*p* < 0.001) lower than in GS patients and only slightly higher than in normal subjects (*p* = 0.04). However, serum-conjugated bilirubin (CB) levels did not significantly change following a caloric reduction in all three patient groups. No subjects with average postfasting serum bilirubin concentrations exhibited hepatic UDP-glucuronyltransferase (UDPG-T) activity levels reduced to those observed in GS, except in a few cases associated with severe liver fibrosis or fatty infiltration. Under the caloric restriction, UDPG-T activity in two patients with hemolysis was reduced to levels observed in GS. According to the authors, the caloric-induced rise in serum unconjugated bilirubin (UCB) is independent of the prefasting bilirubin level. Furthermore, the dietary test may be diagnostically informative even in patients with GS and normal prefasting serum bilirubin levels.

In subsequent studies, Felsher [26] compared changes in bilirubin concentration in four patients with Gilbert’s Syndrome (GS) following a low-calorie and normocaloric diet with significantly reduced carbohydrate, protein, and fat content. For the first two days, patients received a balanced baseline diet (2300 to 2500 cal: 260 g carbohydrates, 90 g protein, 100 g fat). On the third day, the energy value of the diet was reduced to 300 kcal (30 g carbohydrates, 20 g protein, 10 g fat). After 2–4 days of this regimen, the baseline diet was reintroduced for four days. Subsequently, three additional isocaloric diets, each matching the baseline diet in calories and lasting for four days, were sequentially administered to three patients in randomly selected order. These diets were low-carbohydrate (30 g carbohydrates, 169 g protein, 178 g fat), low-protein (22 g protein, 370 g carbohydrates, 93 g fat), or low-fat (10 g fat, 444 g carbohydrates, 118 g protein). Additionally, two patients received a high-fat (220 g), low-carbohydrate (49 g), and low-protein (52 g) isocaloric diet. One patient exclusively received a low-carbohydrate and low-protein isocaloric diet. Each morning before breakfast, blood samples were collected from patients to measure serum total bilirubin (TB) and conjugated bilirubin (CB) concentrations. Significantly higher TB concentrations were observed after the low-calorie diet than the balanced diet (2.8 vs. 1.3 mg/dL). Moreover, TB levels after different isocaloric diet variations with reduced macronutrient content were, on average, 1.1 mg/dL lower than after the low-calorie diet. Low-protein and low-carbohydrate isocaloric diets did not increase serum TB concentration. None of the diets influenced CB concentration in the blood. Additionally, patients’ body mass changed only after applying the low-calorie diet. The author concluded that the hyperbilirubinemia in individuals with GS is primarily due to caloric restriction itself, which impairs bilirubin glucuronidation capacity in the liver by reducing UDP-glucuronic acid formation, rather than changes in diet composition, as neither negative nitrogen balance nor reduced lipid content had any impact on bilirubin levels.

Based on the premise that bile acids have superior diagnostic utility to aminotransferases in certain liver diseases, Briheim et al. [27] conducted a study in the 1980s to determine the serum concentrations of these acids in patients with Gilbert’s syndrome (GS) (n = 12, both genders), depending on their varying bilirubin levels. The serum concentrations of cholic acid, chenodeoxycholic acid, and unconjugated bilirubin (UCB) were measured in patients after overnight fasting. Subsequently, for three days, patients consumed a standardized low-calorie liquid test meal (1.7 MJ/24 h, approximately 406 cal, 50 g fat), and postprandial bile acid levels were measured. Following caloric restriction, a characteristic increase (average of 140%) in serum UCB levels was observed in GS patients. The 150 min values of cholic and chenodeoxycholic acid were slightly higher after reduced caloric intake (*p* < 0.02 and *p* < 0.05, respectively), but no significant differences were found between values obtained before and after caloric restriction. However, fasting and postprandial bile acid concentrations remained within the reference range before and after the reduced caloric intake. No significant correlation was found between the increase in UCB and postprandial bile acid concentrations. According to the authors, different mechanisms are involved in the hepatic uptake and secretion of bile acids and bilirubin. Therefore, measuring serum bile acid concentrations can assess liver function in GS patients independently of the degree of hyperbilirubinemia. Their normal levels exclude liver function disorders, allowing avoidance of more invasive diagnostic methods such as liver biopsy.

Ricci and Ricci [28] hypothesized that food in the intestines controls serum bilirubin levels. They conducted a study on individuals with Gilbert’s syndrome (GS) (n = 18, both genders) and a control group (n = 10, both genders). In Diet A, caloric restriction involved providing patients with 400 cal/24 h in the form of 100 g sucrose. Conversely, in Diet B (volumetric), patients were given 250 g of wheat bran with 100–250 mL of barium sulfate solution, divided into 2 or 3 meals. Diet B provided 1674 kJ (400 cal: 95% carbohydrates, 0.5% fats, and 4.5% proteins). To enhance palatability, patients were allowed to drink tea (500 mL) and water ad libitum, with permitted artificial flavors and saccharin. Blood samples were taken from the patients before the administration of Diet A, after 24 h, and after a minimum interval of 2 weeks, during which the patients were on Diet B. Serum total bilirubin (TB) levels were measured. In healthy individuals, the increase in plasma TB levels was comparable after both diets (A and B) and was not statistically significant (Diet A from 11.1 to 23.4 µmol/L; Diet B from 10.1 to 18.1 µmol/L). However, in individuals with GS, TB levels significantly (*p* < 0.001) increased to a greater extent after Diet A compared to the control group (from 34.1 to 81.4 µmol/L) than after the volumetric Diet B (from 33.9 to 65.2 µmol/L). The serum bilirubin concentration results from a balance between the production rate and the hepatic clearance capacity. The TB levels did not differ significantly between the study groups depending on the diet applied. According to the authors, an intestinal factor, possibly a hormone whose release is dependent on the presence of a large amount of food, plays a role in controlling caloric restriction-associated hyperbilirubinemia.

Gentile et al. [29] compared the diagnostic efficacy of two tests: nicotinic acid (NA) and reduced caloric intake (fasting/hyperbilirubinemia, FH) at 400 calories regarding specificity, sensitivity, and accuracy for optimistic prediction. The authors conducted the tests on 40 patients with Gilbert’s syndrome (GS) (both genders) and 20 healthy controls (both genders). Patients with GS were divided into two groups: GS1 (10 males and 10 females) with high serum bilirubin and GS2 (13 males and 7 females) with average values. In the first test, patients received an intravenous injection of 5 μmol/kg BW NA, and blood was drawn at 0, 30, 45, 60, 120, 180, and 240 min post-injection to measure UCB and CB levels. In the second test, after three days of a normocaloric diet, patients were given a restricted diet of 400 calories for 48 h with an allowance for moderate physical activity. Blood samples were collected at 0, 24, and 48 h, and UCB levels were measured within 30 min of collection. It was found that before the tests, the mean serum UCB level in GS1 patients was significantly (*p* < 0.001) higher than in the control group (23.17 vs. 10.55 μmol/L). However, no such difference was observed between GS2 and the control group (13.12 vs. 10.55 μmol/L). Considering gender, GS1 males had significantly (*p* < 0.05) higher UCB levels than females (28.04 vs. 18.30 μmol/L), whereas no gender difference was observed in the control group (10.99 vs. 9.81 μmol/L) or GS2 (14.12 vs. 12.78 μmol/L). After the NA test, a statistically significant difference (*p* < 0.001) was observed between GS and controls in UCB bilirubin levels, the maximal increment, the AUC, and the retention at 240 min. A significantly higher hyperbilirubinemic effect was also present in GS2 subjects, with no difference observed between GS1 and GS2. There were no gender differences in retention at 240 min. However, significant differences (*p* < 0.001) were noted between males and females with GS in both AUC (6721 vs. 4050 μmol/L/240 min) and the maximal increment (*p* < 0.02) of UCB (60.57 vs. 42.81 μmol/L). In the reduced caloric intake test, UCB bilirubin was significantly higher in GS1 than in GS2 and controls, with no difference in controls. Serum UCB rose significantly (*p* < 0.001) in GS1, GS2, and normal subjects after 24 h of caloric restriction, but not after 48 h. Considering sex, UCB concentration after 24 h was significantly (*p* < 0.05) higher in men with GS than in women (61.51 vs. 38.51 μmol/L), and (*p* < 0.01) after 48 h (78.31 vs. 47.58 μmol/L). In the control group, no gender differences in UCB concentration were found. The authors concluded that in the diagnosis of GS, hyperbilirubinemia induced by NA, particularly its concentration at 240 min post-administration, was more effective for both genders than fasting-induced hyperbilirubinemia.

In the 1990s, Kapıcıoğlu et al. [30], considering that the Muslim community forms about one-third of the world’s population, conducted a study on the impact of the mandatory 30-day fast during Ramadan on unconjugated hyperbilirubinemia in adults with Gilbert’s syndrome (GS) (n = 10). Before the study, the levels of total bilirubin (TB), unconjugated bilirubin (UCB), and conjugated bilirubin (CB) in serum were measured in the patients. During Ramadan, the patients consumed meals at 4:00 AM and 8:00 PM, totaling approximately 2500 calories (approximately 30 g of carbohydrates, 20 g of protein, and 10 g of fat). After measuring bilirubin levels at 4:00 AM, the patients did not eat or drink for 16 h for religious reasons. Throughout the 4-week fasting period, bilirubin levels were measured weekly in the patient’s serum. The authors found that after the first day of fasting, all patients with GS showed a significant (*p* < 0.013) increase in TB and UCB levels compared to pre-fasting levels (1.4 and 2.6 vs. 1.0 and 2.1 mg/dL, respectively). In contrast, the CB level remained similar (0.4 mg/dL). Following additional days of fasting (7, 14, and 30 days), the levels of TB and UCB decreased to pre-fasting values and did not change after that. The CB level remained consistent throughout the fasting period. The authors concluded that the results could be significant for diagnosing GS and its clinical features within the Muslim population.

Rodrigues et al. [31] conducted a study to evaluate the effect of Hb concentration and non-genetic factors (smoking, health status, alcohol consumption, physical activity, oral contraceptive therapy, fasting time, and caloric intake) and the genetic contribution of UGT1A1 polymorphisms on bilirubin levels in 146 young (20.7 ± 2.6 years) Portuguese women to eliminate the potential interference of age and sex. Hematological parameters were measured in their blood, and bilirubin levels were measured in plasma. A screening for the TA duplication in the UGT1A1 gene was conducted. Anthropometric measurements were taken, and body fat content was determined using bioelectrical impedance analysis. Data on fasting time, smoking, oral contraception, and energy intake were obtained from questionnaires filled out by the participants. Physical activity was assessed using the Short Form of the International Physical Activity Questionnaire. The participants were divided into three groups according to tertiles of blood bilirubin concentration (≤6 μmol/L, n = 49; 6–9 μmol/L, n = 49; ≥9 μmol/L, n = 48). The hematologic studies showed that subjects in the second and third tertiles had significantly higher Hb concentration, hematocrit (Ht), mean corpuscular hemoglobin (MCH), and mean corpuscular hemoglobin concentration (MCHC) and lower platelet counts, along with an increased frequency of homozygosity for the c.-41_-40dupTA allele, compared with those in the first tertile. No significant differences were found between groups for total and differential white blood cell counts. It was found that women in the third tertile had a significantly higher red blood cell (RBC) count compared to the first tertile. There were no significant differences between groups in physical activity, smoking, oral contraception, body fat, alcohol consumption, and fasting time. A significantly (*p* < 0.009) lower BMI was found in the third tertile compared to the first. Significant positive correlations were observed between total bilirubin (TB) concentration and Hb, Ht, MCH, and MCHC. The c.-41_-40dupTA allele (genetic factor), Hb, BMI, and fasting time were identified as the main factors associated with bilirubin levels in the blood. According to the authors, since the primary source of bilirubin is the degradation of hemoglobin (Hb) in plasma by macrophages from the reticuloendothelial system, it can serve as a diagnostic marker for Gilbert’s syndrome (GS), as an increase in its blood concentration follows an increase in bilirubin levels (≥6 μmol/L). Additionally, increased red cell mass can be a clinical manifestation of GS or its exacerbation. The hematologic studies showed that subjects in the second and third tertiles had significantly higher Hb concentration, hematocrit (Ht), mean corpuscular hemoglobin (MCH), and mean corpuscular hemoglobin concentration (MCHC) and lower platelet counts, along with an increased frequency of homozygosity for the c.-41_-40dupTA allele, compared with those in the first tertile. No significant differences were found between groups for total and differential white blood cell counts. It was found that women in the third tertile had a significantly higher red blood cell (RBC) count compared to the first tertile. There were no significant differences between groups in physical activity, smoking, oral contraception, body fat, alcohol consumption, and fasting time. A significantly (*p* < 0.009) lower BMI was found in the third tertile compared to the first. Significant positive correlations were observed between total bilirubin (TB) concentration and Hb, Ht, MCH, and MCHC. The c.-41_-40dupTA allele (genetic factor), Hb, BMI, and fasting time were identified as the main factors associated with bilirubin levels in the blood. According to the authors, since the primary source of bilirubin is the degradation of hemoglobin (Hb) in plasma by macrophages from the reticuloendothelial system, it can serve as a diagnostic marker for GS, as an increase in its blood concentration follows an increase in bilirubin levels (≥ 6 μmol/L). Additionally, increased red cell mass can be a clinical manifestation of GS or its exacerbation. The authors found that fasting time was an independent variable related to the concentration of TB. In the fasted state, subjects with average bilirubin conjugation capacity might have higher intestinal bilirubin accumulation compared to those with low bilirubin conjugation capacity due to the UGT1A1 polymorphism, which explains the higher bilirubin concentration in women with average TA counts and UGT1A1 repeats and polymorphisms, given the same fasting time. The authors suggest that studies should be conducted to determine the effects of these factors at different ages in both men and women and to investigate whether UGT1A1 changes, other than TA duplication in the promoter region, may affect TB levels.

In the last decade, Mölzer et al. [32], as part of the BiliHealth project, attempted to elucidate metabolic differences and altered energy regulations between healthy individuals and those with GS in response to fasting. The study focused on the AMPK pathway, which integrates and defines energy and macronutrient metabolism at both the cellular and whole-body levels. The study group consisted of 120 individuals, both healthy and diagnosed with GS (20 men and 40 women each), divided into two age groups: <35 years (n = 66, including 33 healthy and 33 with GS) and >35 years (n = 54, including 27 healthy and 27 with GS). On the day before the study, all participants underwent a 16 h fast, were given a 400-calorie diet, and had blood drawn to measure UCB levels (</≥17.1 μM) to confirm the presence of GS. Blood tests included liver enzymes, hormones, lipid profile parameters, and UGT1A1 genotyping. Additionally, anthropometric measurements, body composition analysis, and a questionnaire on the weekly frequency of healthy food consumption, snacking, red meat intake, and alcohol consumption were completed by the participants. Significant (*p* < 0.000) differences were found between the groups in blood UCB levels and the distribution of TA repeats (i.e., UGT1A1*28 genotype). GS patients did not significantly differ in their intake of healthy foods, snacks, red meat, alcohol, or physical activity compared to the control group. No differences were observed in liver health parameters and iron levels (AST, ALT, γ-GT, LDH, albumin, transferrin, and ferritin) between the groups. However, GS patients had significantly lower BMI (22.8 vs. 25.4 kg/m2), blood glucose levels (81.0 vs. 86 mg/dL), insulin (4.1 vs. 5.9 μU/mL), C-peptide (1.2 vs. 1.4 ng/mL), and triacylglycerols (73 vs. 85 mg/dL), indicating that they are leaner and healthier and thus less prone to metabolic diseases or premature death. In GS individuals, compared to the control group, levels of p-AMPK α1/α2, PPAR α/γ, and PgC1α were significantly (*p* < 0.001) higher (respectively: 178; 400; 1401; 206 vs. 112; 292; 1080; 165 rfU), indicating an increased metabolic response to fasting. However, no difference was observed between the groups in AMPKα1 gene expression. The authors provided evidence that GS’s energy and macronutrient metabolic response to fasting is markedly increased. Consequently, although all subjects were metabolically healthy and within reference ranges for blood biochemistry parameters, several intergroup differences confirmed the better metabolic health of individuals with GS. Serum UCB increased in response to fasting and was found to be the primary determinant of post-translational activation of AMPK α1/α2 and BMI.

Vajro et al. [33], hypothesizing that GS can be transferred from a donor to recipients of orthotopic liver transplants (OLT), investigated the prevalence of this syndrome in pediatric and adolescent recipients (pre-pubertal), a group in which the influence of hormonal factors on bilirubin UDPGT levels is relatively low and rarely associated with clinical symptoms. The study involved 46 patients in the pediatric ward for a year and had undergone transplantation an average of 3.7 years prior (range 0.5–5 years). The average follow-up time was 5.5 years (range 1.0–9.0 years). The control group consisted of 20 individuals without OLT and GS. Serum bilirubin levels and its fractions, monoglucuronide, and sex hormones were measured in OLT patients. The 2-bp (TA) insertion in the promoter of the gene coding for the enzyme bilirubin glucuronosyltransferase associated with GS was screened. Out of the 46 OLT patients, 4 (2 girls, 2 boys, mean age 3.1 years, range 1.2–7 years, 9% of subjects) showed hyperbilirubinemia, with TB levels ≥ 1.3 mg/dL and normal CB levels (<0.3 mg/dL). In three children, hyperbilirubinemia was detected immediately after OLT, while in one child, it was detected a year later and persisted throughout the observation period (range 2.0–8.1 years). Following several tests involving one-day reduced calorie intake and prolonged overnight fasting, TB levels in these patients’ blood samples increased, while CB levels remained unchanged. Returning to a normocaloric diet caused both bilirubin levels to drop to baseline. Significantly lower TB levels (1.2 mg/dL, *p* < 0.01) were observed in OLT recipients without hyperbilirubinemia (n = 20) when the same test was conducted. High relative amounts of UCB IXa and a predominance of monoglucuronide over diglucuronide, characteristic of GS, were also observed. DNA analysis of liver donor lymphocytes (four men) showed that they were either homozygous for the TA insertion allele, homozygous for the normal allele, or heterozygous for the TA insertion allele. Hematological parameters and levels of LDH and sex hormones in all patients were within the normal range. The likelihood of developing GS was significantly higher (*p* = 0.01) in pediatric OLT recipients (regardless of sex) who received a liver from an adult donor. According to the authors, the prevalence of GS in pediatric OLT recipients may be higher than expected in the average pre-pubertal population and may present an atypical early form. Results from the HPLC analysis of serum bilirubin fractions, TB levels, and CB ratios after just one dietary test could allow for a rapid diagnosis of GS, while molecular data could confirm this diagnosis.

### 3.2. Consumption of Various Diet Variants, Vegetables, Fruits, and Alcohol in Gilbert’s Syndrome

Among the selected publications, seven focused on consuming various diet variants, vegetables, fruits, and alcohol in Gilbert’s Syndrome (Table 2). The studies included different diet variants, namely, standard, restrictive, and hypercaloric, as well as the consumption of six botanical groups of vegetables and fruits, various quantities of vegetable and fruit servings, post-bariatric surgery diet, and alcohol consumption. The studies were conducted in groups of healthy patients stratified by genotype, age, gender, and ethnicity. The duration of the diets ranged from two days to over four years of observation.

In the 1970s, Gollan et al. [34] hypothesized, based on contemporary studies, that dietary factors and fasting could influence the increase in blood bilirubin levels. They conducted a study to determine whether unconjugated hyperbilirubinemia in patients with Gilbert’s syndrome arose due to the nature of the diet or the route of administration and to assess the relationship between these factors and fasting-induced hyperbilirubinemia. The experiment was conducted on 29 patients (both genders) aged 14–46 years, who were given a normal diet (I) with an energy value of 10 MJ (2400 cal) before the study, and their plasma total bilirubin (TB) concentration was measured. Patients were then given six test diets: (II, n = 8) low-energy, standard (1.6 MJ, 400 cal); (III, n = 5) low-energy, standard + IV glucose (10 MJ, 2400 cal); (IV, n = 5) low-energy, standard + IV lipid (8.4 MJ, 2000 cal); (V, n = 7) high-carbohydrate, low-lipid (fluid) (10 MJ, 2400 cal); (VI, n = 4) high-carbohydrate, reduced-lipid (10 MJ, 2400 cal); (VII, n = 5) low-energy, high-lipid (1.6 MJ, 400 cal). The fluid diet contained only 2 g of lipids (0.6% of total energy intake) and mainly consisted of glucose polymer, fruit juice, and skim milk. After the test period, patients resumed a normal diet, and their blood TB levels were measured for one or two days. The mean TB concentration in the blood of GS patients before the normal diet was comparable to the TB concentration after 2 days of the diet (34.0 vs. 31.1 μmol/L, respectively), with no diurnal variations. The highest increase in TB concentration was observed in patients after diet II (low-energy 400 cal) and diet III (low-energy with IV glucose 2400 cal), with increases of 135% and 127%, respectively. The high-carbohydrate fluid diet resulted in a 76% increase in blood TB levels in patients, whereas the low-energy, high-lipid diet led to a 49% increase. The lowest increase in blood TB concentration was observed with the high-carbohydrate, reduced-lipid diet (8%), which was similar to the normal diet. The level of hyperbilirubinemia achieved after oral carbohydrate administration was significantly (*p* < 0.025) lower than that after intravenous glucose administration or the standard low-energy diet (*p* < 0.001). The low-energy, high-lipid diet (VII) significantly (*p* < 0.001) reduced hyperbilirubinemia to levels lower than those observed with the standard 400-calorie diet. According to the authors, excluding lipids from the diet in some patients receiving parenteral nutrition or during the postoperative period may explain the occurrence of unconjugated hyperbilirubinemia. Furthermore, additional research on neonatal hyperbilirubinemia is warranted, as some initial feeding regimens low in lipids could exacerbate hyperbilirubinemia. Therefore, it is advisable to provide diets with a small amount of lipids to preterm infants/newborns with jaundice.

Peterson et al. [35], in their studies, assumed that UGT1A1 also conjugates 17β-estradiol (the most biologically active estrogen) and estriol, as well as exogenous compounds such as phenols, anthraquinones, and flavones, many of which are found in the diet. The polymorphisms in UGT1A1 may influence bilirubin clearance, endogenous estrogenic load, and exposure to xenobiotics (as well as heterocyclic amines and polycyclic aromatic hydrocarbons). Animal model studies have shown that biologically active vegetable substances (such as sulforaphane, protein and soy isoflavones, dietary monoterpenes, and allyl sulfides) increase UGT activity in the liver. Therefore, the authors evaluated the impact of consuming foods from the botanical families *Cruciferae* (e.g., broccoli), *Rutaceae* (citrus fruits), *Liliaceae* (e.g., onion), and *Leguminosae* (legumes) on UGT activity in humans by measuring blood bilirubin levels, depending on genotype. The recruitment criterion for the study was the consumption of 2.5 or 4.5 servings per day of *Cruciferae* and *Leguminosae.* The study was conducted on three individuals (both sexes, aged 19–40 years) and included demographic data, health history, a 3-day food record, blood TB and CB measurements, and UGT1A1 promoter genotype determination. Vegetable consumption from the four botanical families was assessed according to standardized portions. Participants were divided into genotype groups 6/6 or 6/7 (n = 169) and 7/7 (n = 22) because previous studies have shown that the greatest difference in UGT1A1 activity is between 7/7 and the presence of one or more 6 alleles; thus, the 6/6 and 6/7 genotypes were combined for the analysis. Total fruit consumption ranged from 0 to 11 servings and vegetables from 0 to 13 servings per day. Consumption of products from the *Cruciferae, Rutaceae*, and *Leguminosae* groups ranged from 0 to 4 servings per day, while Liliaceae ranged from 0 to 2. There were significantly (*p* < 0.001) higher TB, CB, and UCB concentrations in men than in women. There was a significant inverse association between BMI and CB (*p* < 0.001). There was no significant difference in the consumption of groups of botanical plants depending on the genotype of the respondents. The authors found no significant associations between TB and intake of carbohydrates, protein, fat, alcohol, total fruit, and vegetables when controlling for UGT1A1 genotype, age, sex, and BMI. Similarly, there was no association between TB, CB, or UCB concentrations in the blood of the subjects and any botanical plant group. However, there was a significant inverse association between TB, CB, and UCB (*p* < 0.017) and the interaction of the UGT1A1*28 genotype with *Cruciferae* intake. There was also a significant inverse association between the UGT1A1-Cruciferae gene interaction and CB (*p* < 0.003) and UCB (*p* < 0.020), but the authors did not present these data. Individuals with the 7/7 genotype had lower blood bilirubin levels with increased consumption of cruciferous vegetables compared to those with 6/6 or 6/7 variants. The authors explained the observed changes by noting that glucosinolates in cruciferous vegetables can be hydrolyzed by the plant enzyme myrosinase to indoles and isothiocyanates when plant cells are damaged (e.g., during cutting or chewing). Indoles bind to AhR (aryl hydrocarbon receptor); sulforaphane induces UGT1A1 by modulating the extracellular signal-regulated kinase mitogen-activated protein kinase pathway in Caco-2 cells. Additionally, although the interaction with cruciferous compounds has not been studied, nuclear receptors, the pregnane X receptor, and the constitutive androstane receptor influence UGT1A1 expression and activity. According to the authors, their study confirmed that individuals with reduced UGT1A1 activity and the 7/7 genotype might, through dietary intervention, have greater potential to reduce the risk of carcinogenesis concerning carcinogens such as heterocyclic amines and polycyclic aromatic hydrocarbons conjugated by this enzyme.

The continuation of the research by Peterson et al. [35] was the study published by Navarro et al. [36]. The authors justified their research by stating that individuals with the UGT1A1*28 polymorphism (characteristic of GS) have reduced transcription compared to the wild type, resulting in decreased enzyme UDP-glucuronosyltransferase (UGT) activity. Such individuals have an increased risk of developing cancer (especially stomach and lung cancer), and chemoprevention is therefore advisable. One group of foods with such properties is cruciferous vegetables (*Brassicaceae*), as the isothiocyanates they contain partially increase the activity of phase II conjugating enzymes, such as UGT. Isothiocyanates, especially GSTM1, are metabolized by glutathione S-transferases, but genetic variants can alter their clearance. In individuals with the GSTM1-null and GSTT1-null genotypes, isothiocyanates are metabolized more slowly, leading to a greater accumulation of isothiocyanates at the tissue level; thus, they are likely to a greater chemoprotective effect. The aims of the study were to determine the effect of cruciferous vegetable consumption and two different doses on bilirubin conjugation as a measure of UGT1A1 activity and to assess the impact of the GSTM1-null and GSTT1-null variant or the UGT1A1*28 polymorphism on this response. A secondary aim of the study was to evaluate whether adding apiaceous vegetables to cruciferous foraging further alters this response. In a study conducted among 70 individuals (aged 20–40 years), the researchers assessed a feeding trial of four diets: (i) a basal diet devoid of fruit and vegetables, (ii) the basal diet supplemented with a prescribed amount of cruciferous vegetables (single-dose cruciferous diet); (iii) the basal diet supplemented with twice this amount of cruciferous vegetables (double-dose cruciferous diet); (iv) and the basal diet supplemented with cruciferous and apiaceous vegetables (single-dose cruciferous plus apiaceous vegetable diet). The single dose of cruciferous vegetables was approximately 7 g/kg body weight, the double dose was approximately 14 g/kg, and the mixed diet included 7 g/kg of crucifers plus 4 g/kg apiaceous vegetables. Diets were first standardized according to servings for a 70 kg individual, determining g food/kg body weight for each plant item. Food was served as meals under the supervision of the research staff or provided for the weekends. Participants kept food diaries, and plate waste was recorded. Buccal cells, collected before randomization, were isolated, and DNA was extracted to determine GSTM1 and GSTT1 genotypes and participant eligibility. Genotyping of the white blood cell DNA for the UGT1A1 polymorphism was conducted. On days 0, 7, 11, and 14 of each feeding period, blood was drawn in the morning after a 12 h overnight fast to measure TB and CB and calculate UCB. On day 13 of each feeding period, participants collected urine for 24 h to assay urinary total isothiocyanates. Before the experimental diets were implemented, no significant differences in demographic parameters were observed between the groups of individuals depending on the UGT1A1 genotype. However, individuals in the group with the UGT1A1*28/28 genotype had significantly (*p* < 0.02) higher levels of TB and CB compared to the other genotypes (UGT1A11/1 and UGT1A11/*28).

The response to all three diets containing vegetables differed statistically significantly from the basal diet, with lower mean TB levels associated with consuming the test plant diets (*p* < 0.02 for all three), suggesting more excellent UGT1A1 enzyme activity. There was also a dose effect of cruciferous vegetables in the diet, as significantly (*p* < 0.03) lower mean TB levels were found in individuals consuming the double dose of cruciferous vegetables compared to the single dose. The same pattern was observed for UCB, but there were no differences in CB; however, the authors did not present these results. The interaction between diet and gender was not significant. Considering the UGT1A1 genotype variant, significant differences in TB levels were found between individuals with *1/*1 (40%) and *28/*28 (28%) on the basal diet (*p* < 0.01) and single-dose cruciferous vegetable diets (*p* < 0.03). The differences between *1/*1 and *1/*28 were statistically significant only on the single-dose cruciferous diet, with *1/*28 having 10% higher bilirubin concentrations than *1/*1 (*p* = 0.02). Comparing the differences in bilirubin response to diets between groups, individuals with *28/*28 responded slightly better to the single-dose cruciferous diet compared to those with *1/*28 (*p* = 0.049) and the apiaceous diet (*p* = 0.03). Although UGT1A1 activity, measured by serum bilirubin levels, was higher in individuals with GSTM1-null compared to GSTM1+ individuals, it was not statistically significant. There was no statistically significant main effect of the combined GSTM1 and GSTT1 genotypes or the genotype-by-diet interaction. Analysis of urine collected from patients on day 13 of the diet indicated a steep and dose-dependent increase in isothiocyanate excretion compared to the period on the basal diet. The sample collection day had a significant (*p* < 0.001) effect, but the interaction of individual diets was not statistically significant. Dietary supplementation with vegetables resulted in lower serum bilirubin concentrations on days 11 and 14 compared to day 7 (*p* < 0.01), but its concentrations slightly increased from days 11 to 14 (*p* = 0.04). The relationship between CYP1A21F and bilirubin was not statistically significant, and the data were not presented. According to the authors, the increased UGT1A1 enzyme activity and reduced serum bilirubin levels in the studied individuals were contributed to by cruciferous vegetables containing a mixture of isothiocyanates and indoles and apiaceous vegetables containing furanocoumarins. Therefore, these findings may impact the metabolism of carcinogens through dietary intervention, particularly among individuals with UGT1A128/*28.

Saracino et al. [37] conducted a study whose aim was to detect the interaction between the genotype and the consumption of citrus fruits, cruciferous vegetables, and soy (and phytochemical components contained in these products), inducing UGT (UDP) activity on the concentration of bilirubin in the blood. Initially, the study was conducted among 293 adults of both sexes (125 women and 146 men aged 20–40 years) living in the Seattle area, but not all completed the study. Participants completed a Food Frequency Questionnaire (FFQ) covering 3 months (n = 274) and provided 3-day food records (n = 289) to assess fruit and vegetable (F&V) intake, categorized by botanical families (*Cruciferae*, *Rosaceae, Solanaceae*, *Leguminosae, Rutaceae*, *Umbelliferae*). *Cruciferae* are rich in sulfur-containing glucosinolates, and the *Solanaceae* and *Rutaceae* families contain carotenoids and many flavonoids. Fruit in the *Rosaceae* family are also abundant in flavonoids, and legumes are rich in lignans. Participants (n = 283) underwent anthropometric measurements (body mass and height), and after a 12 h fasting period, blood was drawn to determine TB, CB, and UCB concentrations and to genotype UGT (UGT1A1 genotype 6/6; 6/7; 7/7) in leukocyte DNA. It was found that gender (men vs. women), race (Asian vs. Caucasian), and UGT1A1 genotype were significantly associated with serum concentrations of all three bilirubins. TB and UCB levels were significantly (*p* < 0.05) lower among non-Caucasian races. There was a linear trend of increasing TB, CB, and UCB (*p* < 0.001) with more UGT1A1*28 alleles. Age (<30 or ≥ 30 years) and duration of physical activity (≤6 or >6 h/week) were not associated with any form of bilirubin concentration. There was no significant association between total fruit and vegetable intake servings (<4, 4–5, and >5 per day) estimated from the FFQ and TB levels. However, among women compared to men, an interaction was found between UGT1A1 genotype and citrus fruit intake (*p* = 0.006) and *Rutaceae* (*p* = 0.03) assessed by 3DFR, related to TB and UCB concentrations. Women with the 7/7 genotype who consumed approximately 0.5 daily servings of citrus fruit or *Rutaceae* had lower (approximately 30%) TB, CB, and UCB concentrations than those who consumed less. The authors explain this via the beneficial effect of phytochemicals (such as quercetin) found in citrus fruits, which increase UGT activity by inducing gene expression. The authors also suggest that women consuming more citrus fruits with phytochemicals had lower serum bilirubin levels than men (regardless of UGT1A1 genotype) due to estrogen and/or progesterone-mediated UGT1A1 transcription. The estrogen receptor (ER)-mediated increases in transcriptional activity of the aryl hydrocarbon receptor (AhR) affect UGT1A1. Recent fruit and vegetable intake (estimated by 3DFR) helps detect associations between fruit and vegetable intake and blood bilirubin levels, as changes in UGT expression/activity can be detected within 1 h of phytochemical exposure. The described studies indicated that some components of fruits and vegetables might increase UGT1A1 activity in individuals with the 7/7 genotype variant, which is most common in GS, thereby potentially improving the clearance of certain carcinogens from the body and reducing cancer risk.

Stojanović and Vukavić [38] assumed that high blood bilirubin levels (due to its antioxidant stimulation of nitric oxide production and its relaxing effect) might decrease the baseline tension of bladder and ureter muscles, potentially leading to urinary retention and stasis, predisposing to urinary tract infections (UTIs). They assessed the influence of hyperbilirubinemia in children with Gilbert syndrome (GS) on their lower urinary tract function. The study included 29 children aged 8–17 years (both sexes) who underwent a diagnostic 24 h fasting test (with zero energy intake and water intake ad libitum), followed by a 24 h hypercaloric intake (30% higher energy intake than average for their age). Blood samples were collected from the children at 0, 12, and 24 h fasting and after 24 h of hypercaloric intake to measure glucose concentration, TB, CB, UCB, and urinary ketones. Uroflowmetry was performed after 24 h of fasting (experimental) and after 24 h of a hypercaloric diet (control). According to the survey, 28 children had no history of UTIs, and 29 had no history of constipation. A bell-shaped uroflowmetry pattern was typical in 69% of the children, while 31% had an abnormal pattern. All patients showed a regular uroflowmetric pattern after 24 h of hypercaloric diet intake. Patients with an abnormal uroflowmetric pattern had higher CB concentrations (11.6 vs. 9.35 µmol/L) after 24 h of fasting than those with a regular pattern (*p* = 0.012). There were no significant differences in TB and UCB concentrations. In children with a standard urinary flow curve (UFC), CB concentrations were significantly lower pre-fasting compared to post-fasting (7.06 vs. 9.35 µmol/L, *p* = 0.022), and glucose levels were significantly higher (4.51 vs. 3.76 mmol/L). Similarly, in children with an abnormal UFC, CB concentrations were significantly lower pre-fasting compared to post-fasting (7.91 vs. 11.60 µmol/L, *p* = 0.008), and glucose levels were higher (4.56 vs. 3.29 mmol/L). The children’s bladder capacity (VC) was normal after fasting and consuming a hypercaloric diet. However, the authors did not present the results of urinary ketone levels in the urine of the children studied. According to the authors, hyperbilirubinemia, with or without hypoglycemia, may cause intermittent dysfunction of the lower urinary tract in children. The significantly higher CB concentrations in patients with abnormal UFC than those with normal UFC after 24 h of fasting require confirmation in a larger patient cohort.

In their study, Huppertz et al. [39] aimed to determine the potential impact of Gilbert syndrome (GS) on the formation of ethyl glucuronide (EtG) as a direct marker of ethanol consumption and a product of glucuronidation, both alone and in combination with ethyl sulfate (EtS), used for monitoring abstinence in individuals. The research involved 30 individuals (both genders, aged 18–71 years) with GS, from whom urine samples were collected after at least 2 days of abstinence from alcohol. Participants were instructed to drink 0.1 L of sparkling wine (9 g of ethanol) within 5 min. Anthropometric measurements were taken from the patients, and they were asked to complete a lifestyle questionnaire, including alcohol consumption behaviors. Urine samples were collected at 3, 6, 12, and 24 h post-ethanol consumption to measure EtG, EtS, and creatinine levels. Blood samples from the patients 3 h after wine consumption were analyzed for morphological parameters, ethanol concentration, TB, CB, and liver enzymes. The BMI of the patients ranged from 15.4 to 27.3 kg/m^2^. Ten individuals declared mainly wine consumption (from one glass to two bottles per week), thirteen preferred beer (from two to ten bottles per week), and only one patient regularly consumed larger quantities (≥2 drinks per day, more on weekends). No significant abnormalities were found in the urogenital system or blood morphology. AST activity was within the normal range for all patients, with one patient having slightly elevated ALT (above the range of 10–50 U/L), and five patients had increased GGTP levels. Elevated TB concentrations were observed in 21 patients, while UCB levels were normal in 9 patients. EtG and EtS were detected in all patients across a wide range of concentrations (EtG: 3 h sample 0.5–18.43 mg/L and 6 h sample 0.67–13.8 mg/L; EtS: 3 h sample 0.87–6.87 mg/L and 6 h sample 0.29–4.48 mg/L). There was no evidence of impaired EtG formation. Mutations in the UGT1A1 isoform in GS do not affect the overall formation of ethyl glucuronide. Therefore, EtG appears to be a suitable marker of ethanol consumption even in individuals with GS. Gilbert syndrome also does not affect the formation of EtS.

Izzy et al. [40] presented intriguing findings from their study assessing long-term outcomes of bariatric surgery beyond 4 years (mean 8.7 years ± 1.4 years) in 10 patients (7 women and 3 men, with a mean age of 58.4 years ± 9.6 years) with compensated liver cirrhosis confirmed by a liver biopsy showing nonalcoholic steatohepatitis (NASH). Among these patients, nine experienced an average total body weight loss (TBWL) of 24 kg (19.2% TBWL before surgery, *p* < 0.001), while one patient had an increase in body weight. Nine patients showed decreased BMI, improved HbA1c levels in those with abnormal glycemia, and reduced total bilirubin (TB) levels. None of these individuals experienced liver decompensation (development of ascites, hepatic encephalopathy, or variceal bleeding), cardiovascular events, or mortality. The MELDNa score (Model for End-Stage Liver Disease incorporating sodium levels) was 9 (±6) for most patients, indicating a low mortality risk (1.9%). In contrast, in the patient with Gilbert syndrome (GS), long-term follow-up after bariatric surgery showed a TBWL of 23.5%, a decrease in BMI of 7.7 kg/m^2^, and a reduction in HbA1c levels by 1.3%. However, despite the decrease in HbA1c, insulin treatment was still necessary. Additionally, there was a 1.4 mg/dL increase in TB levels. The MELDNa score for this patient was 14, reflecting a 6% mortality risk.

According to the authors, bariatric surgery in patients with compensated liver cirrhosis can lead to beneficial and sustainable weight loss, improvement or stabilization of coexisting metabolic diseases, and stable liver function, even many years post-surgery. The benefits achieved outweigh the surgical risks in such patients.

## 4. Discussion

Caloric restriction/fasting is a component of human nutrition. However, in the case of Gilbert syndrome, it has primarily been used as a diagnostic tool (provocative tests) for this condition. Studies have shown a correlation between caloric intake and hyperbilirubinemia (impairing the liver’s ability to glucuronidated bilirubin by reducing the formation of UDPGA—uridine diphosphoglucuronic acid) and fluctuations in serum bilirubin levels in individuals with GS [22]. Reducing energy intake to 300–400 cal/day, which led to increased TB and UCB levels in patients, allowed for the differentiation of GS patients from those with other liver diseases [23]. Researchers attempted to understand the physiological mechanism behind these changes. It was found that changes in bilirubin levels were not due to increased heme turnover [24] or bile acid levels [27] as they remained within reference ranges regardless of the degree of hyperbilirubinemia in GS patients. Subsequent studies aimed to determine whether changes in macronutrient composition, aside from caloric restriction, could contribute to hyperbilirubinemia. However, no such effects were observed when considering nitrogen balance and lipid metabolism [26]. However, a low-energy, high-lipid diet was found to reduce hyperbilirubinemia to levels lower than those caused by the standard 400 cal/day diet [34].

Since caloric restriction (400 cal/day) proved to be a critical diagnostic element for GS, it was found that the form of caloric intake affected the degree of hyperbilirubinemia. The increase in TB levels was more significant after sucrose administration compared to a volumetric meal (bran), suggesting that gastrointestinal hormones influenced hepatic clearance in this context [28]. It was essential to establish the duration of caloric restriction necessary to maintain hyperbilirubinemia (measured by TB and UCB levels). It was shown that TB and UCB levels were significantly higher in the first few days of fasting compared to before fasting [25], while CB levels remained similar. Extending the fasting period up to 30 days (16 h/day during Ramadan) did not result in significant changes in bilirubin fractions [30]. In the search for more sensitive tests for GS diagnosis [29], a nicotinic acid test was used in addition to caloric restriction. This test proved to be more effective (in both genders) at inducing hyperbilirubinemia, particularly at 240 min after drug administration, compared to fasting-induced hyperbilirubinemia following caloric restriction (400 cal/day).

Before the introduction of molecular diagnostics for GS, provocative tests were performed, including caloric restriction, nicotine, phenobarbital, rifampicin, or the “inverse starving test” (after a standard European lunch). However, these tests are now historically significant as they are characterized by low sensitivity and specificity [41,42]. Fasting for 24 h and reducing daily caloric intake by 300 calories, as well as the rifampicin test administered after 2 or 4 h, which shows a significant increase in unconjugated bilirubin (UCB), suggest GS-related hyperbilirubinemia [43]. In healthy individuals, total bilirubin concentration increases by about 60% after 48 h of fasting, whereas in those with GS, its concentration increases by about 90% after 24 h, and UCB concentration increases by over 110% after 24 h [3]. Other methods for diagnosing GS include measuring bilirubin kinetics after intravenous injection of cold crystalline pigment or radiolabeled bilirubin or computing via mathematical methods from the survival of 51Cr-labeled erythrocytes, which provides the most reliable documentation of impaired bilirubin clearance [30].

Additional diagnostics for GS include the invasive procedure of percutaneous liver biopsy with evaluation of the biopsy specimen, although this is not routinely recommended [16]. Histological examination typically reveals pathognomonic changes such as flattening and damage to the microvilli on the vascular pole of hepatocytes, mitochondrial polymorphism, and an increase in endoplasmic reticulum and glycogen. Histopathology typically reveals non-specific lipofuscin pigment within the centrilobular region of the biopsy specimen; otherwise, histology is normal. Currently, the diagnosis of GS is definitively confirmed by molecular testing showing the presence of mutations in the UGT1A1 gene. It should be emphasized that in the case of isolated hyperbilirubinemia, it is essential to exclude hemolytic anemia before making a final diagnosis of liver-origin hyperbilirubinemia [44,45]. Such diagnostic rigor is particularly warranted in cases where GS is familial. Furthermore, other tests such as abdominal ultrasound, hepatobiliary scintigraphy, cholangiography, and/or cholecystography do not reveal abnormalities in individuals with GS compared to healthy individuals [16].

A broader aspect of nutritional status and dietary habits, combined with blood morphological parameters and genetic studies, was first considered in women with GS in the studies by Rodrigues et al. in 2012 [31]. They found that the c.-41_-40dupTA allele (genetic), HGB, BMI, and fasting duration were the main factors associated with blood bilirubin levels. HGB can be a diagnostic marker in GS, as its increase in the blood follows the rise in bilirubin levels. Interesting findings regarding the metabolic health of individuals with GS were also obtained by Mölzer et al. [32], who demonstrated that people with GS had lower BMI, lower blood glucose, insulin, C-peptide, and triacylglycerol levels compared to healthy controls and were less prone to metabolic diseases or premature death. This positive aspect of metabolic health in GS individuals can be explained by the fact that bilirubin is a physiologically important antioxidant and might assist in neutralizing ROS (reactive oxygen species) and preventing oxidative damage. Wagner et al. [46] demonstrated that in 119 individuals, there were significantly higher serum unconjugated bilirubin (UCB) concentrations, lower BMI, 37% higher antioxidant potential as assessed by iron reduction capacity potential (FRAP), elevated advanced protein oxidation products, and lower levels of apolipoprotein B, hs-C-reactive protein, interleukin 6, and interleukin 1 beta compared to 119 healthy individuals. Unconjugated bilirubin might also have anti-genotoxic potential by preventing oxidative damage to DNA [47]. Interestingly, studies have shown a reduced risk of obesity and its health complications [48], i.e., cardiovascular diseases (CVD) such as ischemic heart disease and hypertension and type 2 diabetes, in individuals with GS, less pronounced pro-inflammatory processes [49,50,51], and lower levels of blood lipids and lipoproteins [50,52,53,54,55,56]. Serum metabolomics analysis reveals increased lipid catabolism in mildly hyperbilirubinemic GS individuals [46]. The reduced cardiovascular risk and mortality may be due to the lower body mass index (BMI) in individuals with GS [53]. Hyperbilirubinemia is also associated with a lower risk of metabolic syndrome and non-alcoholic fatty liver disease (NAFLD) and may protect against the development of non-alcoholic steatohepatitis (NASH) [57,58].

Moreover, mild unconjugated hyperbilirubinemia may have beneficial effects, such as reducing the risk of endometrial cancer, lung cancer, breast cancer, colorectal cancer, and Hodgkin’s lymphoma, as well as decreasing cancer-related mortality, owing to the antioxidant and protective properties of bilirubin [2,12,59,60,61]. Considering the impact of intestinal microbiota composition on decreased colorectal cancer risk, Zöhrer et al. [62] found no significant differences between 45 individuals with Gilbert’s syndrome (GS) and 45 healthy individuals. However, they observed greater bacterial diversity among women. Interestingly, in men with Gilbert syndrome (GS), who chronically have elevated unconjugated bilirubin (UCB) levels in their blood, longer telomeres have been observed, which are potential biomarkers of cellular aging, apoptosis, and age-related diseases [63].

Taking into account the positive impact of mild hyperbilirubinemia on reducing the risk of diseases related to oxidative stress, scientists are conducting studies on inducing “Iatrogenic Gilbert syndrome” using drugs (such as probenecid, rifampicin, valproic acid, osimertinib, atazanavir, biliverdin), as well as nutraceuticals (phycobilins from plants, algae, and cyanobacteria) that reduce the activity of hepatic glucuronidation [63,64].

Research by Vajro et al. [33] also underscores that caloric restriction and genetic testing, such as analysis of the 2-bp (TA) insertion in the promoter of the bilirubin glucuronosyltransferase enzyme gene, can be valuable tools for early detection in children and adolescents before puberty of the risk of transmitting GS from donor to liver transplant recipients. Currently [63], the diagnosis of GS includes the following: isolated, asymptomatic unconjugated hyperbilirubinemia (17–70 μmol/L [1–4 mg/dL]); liver enzyme activities (ALT, AST, ALP, GGT) within physiological range; absence of overt signs of hemolysis (reticulocyte count <1.5%, negative Coombs test); and genetic testing reserved for individuals with higher serum bilirubin concentrations to differentiate GS from Crigler–Najjar syndrome type 2 or when considering treatment with medications that affect UGT1A1 activity.

In other studies, attention was drawn to caloric restrictions and their impact on hyperbilirubinemia, lipid metabolism, and vitamin levels. Kamal et al. [3] conducted a screening survey among 1249 Egyptians (aged 14–45 years) and identified 101 (8.016%) individuals with GS, including 83 with GS and 18 with GS after excluding HCV. All reported episodes of jaundice lasted, on average, 19–21 days. Among adult Egyptians with GS, provocative tests (fasting 300 cal/day; with rifampin), nutritional status assessments, UGT1A1 genotyping, liver function tests, and measurements of blood levels of vitamins B12, folate, and D3 were conducted. A two-week diet was implemented, and bilirubin levels were measured on days 0, 7, and 14 of the diet. Folate and vitamin D3 levels were lower in individuals with GS compared to the control group, whereas B12 did not differ significantly. Individuals with GS had significantly lower levels of VLDL and IDL. Fasting > 12 h and a diet < 1200 cal significantly influenced the occurrence of jaundice episodes, while a high-fat diet did not.

Kiranmayi et al. [65] presented interesting data on dietary faddism. This term refers to a specific food or food group that is either overly emphasized or omitted in the regular diet for treating a particular condition. The study focused on the impact of dietary trends among 125 patients (both genders) from India diagnosed with GS, characterized by consistently elevated unconjugated bilirubin (UCB) levels with normal liver transaminases, ceruloplasmin, and serum hemoglobin, without liver discomfort or other chronic diseases. Initially, over 6 months (monthly), the study monitored blood levels of vitamin B12 before and after treatment with cyanocobalamin injections. Vitamin B12 deficiency (megaloblastic anemia) was found in 21 patients as a result of dietary restrictions stemming from nutritional myths, leading to ineffective erythropoiesis and prolonged hyperbilirubinemia. The requirement of methylcobalamin explained the occurrence of anemia in individuals with GS as a cofactor in methionine synthase for synthesizing pyrimidines and purines. Folic acid, essential for this process, transfers a methyl group from methyl-tetrahydrofolate to homocysteine, forming methionine and tetrahydrofolate. Disruption of this reaction leads to megaloblastic anemia. Ineffective erythropoiesis exacerbates existing indirect hyperbilirubinemia in GS patients due to hemolysis. Methylmalonyl-CoA mutase converts methylmalonyl-CoA to succinyl-CoA, requiring 5-deoxyadenosylcobalamin as a cofactor. Defects in this reaction lead to the accumulation of methylmalonyl-CoA, believed to be responsible for the neurological consequences of vitamin B12 deficiency. Symptoms observed in GS patients included fatigue and limb paresthesia. Administering vitamin B12 injections for a month improved blood parameters and reduced the occurrence of neurological symptoms.

In the studies included in this systematic review, significant and practical findings were reported by Peterson et al. [35], Navarro et al. [36], and Saracino et al. [37]. These researchers identified the impact of consuming fruits and vegetables containing various biologically active compounds from different botanical groups on serum bilirubin levels and the ability to enhance UDP-glucuronosyltransferase (UGT) enzyme activity in the liver through gene expression induction, taking into account genetic differences among patients. Their results indicate that the consumption of vegetables from the *Cruciferae* and *Apiaceous* groups and fruits from the *Rutaceae* group by individuals with reduced UGT1A1 activity and the 7/7 genotype may increase UGT enzyme activity in the liver. Such dietary interventions may also reduce the risk of carcinogenic factors associated with this enzyme, such as heterocyclic amines and polycyclic aromatic hydrocarbons. However, the observed effect, characterized by a decrease in serum bilirubin levels, depends on the recommended dose of plant consumption, with the double dose of vegetables proving most effective, along with the appropriate timing of consumption.

From the studies included in the review, several weaknesses emerged, such as older studies relying on observation without a control group; inconsistent criteria for inclusion and exclusion; varying durations of dietary interventions; selection bias in patient groups based on gender, age, BMI, and coexisting conditions, thus potentially not producing representative samples; limited groups with the typical GS genotype (UGT1A1*28 7/7); lack of consideration for ethnic groups; discrepancies between reported food intake by patients and actual consumption, with more accurate reflection in 3-day records than in food frequency questionnaires (FFQs); inability to accurately estimate phytochemical intake due to incomplete data in nutrition databases; consumption levels of vegetables from *Cruciferae* and *Apiaceous* plants, e.g., 5–10 servings per day (~300 g–1300 g), generally not met by residents in various regions worldwide; and the extensive but sometimes conflicting data on the mechanisms of plasma bilirubin response to caloric deprivation.

From the studies included in this systematic review, specific strengths have emerged: newer studies employed a control group and specified criteria for inclusion and exclusion; there was homogeneity among participant groups regarding non-dietary factors influencing UGT enzyme activity such as age, gender, ethnic group, BMI, alcohol and tobacco consumption, dietary supplements and medications, pregnancy or lactation, known allergies/intolerances, weight changes exceeding 4.5 kg in the past year, significant changes in eating habits within the past year (e.g., adoption of a faddish diet), antibiotic use within the past 3 months, food preferences that could affect participation in feeding trials, and other medical conditions; a wide range of fruits and vegetables consumed by participants based on portion size criteria was considered; in some studies, the duration for each tested diet exceeded 2–3 days; blood samples were taken during tested diets, and analysis of multiple blood parameters (morphological, biochemical) and genotypes better illustrated the impact of the applied dietary intervention. Therefore, we agree with Vitek [63] that bilirubin concentrations in both the general population and in people with GS change not only depending on gender, ethnicity, age, smoking, circadian rhythms, seasonal periods, and physical activity but also on nutritional influences.

Therefore, in our opinion, further research should focus on the following: (1) identifying food compounds that could regulate bilirubin levels in individuals with GS by influencing the expression of the hepatic UGT enzyme gene; (2) determining whether UGT1A1 variations other than the TA duplication in the promoter region can affect total bilirubin levels in blood; (3) investigating other types of food that may influence the conjugation of carcinogenic factors in individuals with the UGT1A1*28 7/7 genotype, aiming to reduce the risk of cancer development. However, such studies should be conducted in larger populations or among recruited patients based on the UGT1A1 genotype, encompassing different ages, genders, and ethnic groups. The existence of differences in levels of certain biochemical parameters depending on biogeographical origin underscores the necessity to establish specific reference ranges for each. Therefore, we agree with Vittek’s opinion [63] that it would be prudent to create international registries compiling research data that describe the impact of nutritional and non-nutritional factors and the relationships between them on the health-related quality of life of patients with Gilbert’s syndrome and/or comorbidities.

## 5. Strength

This is the initial systematic review of nutrition in Gilbert’s syndrome, which spanned up to 60 years and was grounded in clinical studies. This review was conducted following the PRISMA guidelines by three independent researchers who evaluated the internal quality of the selected reports. The findings of this review predominantly include randomized clinical trials, thus boasting excellent reliability and serving as the gold standard for assessing intervention efficacy. Moreover, applying the Jadad scale in this review provides an objective framework, minimizing subjective biases.

Considering the precision and reliability in assessing the methodological quality of the studies, we employed the five-point JADAD scale. Our findings indicate that among the reviewed randomized controlled trials, nine scored 2 or lower, seven were rated as medium quality (scoring 3), and three achieved a score of 4, indicating high-quality clinical trials. None of the studies reached the maximum of 5 points on the JADAD scale. This underscores the imperative for future studies to adopt transparent and well-defined methodologies to ensure the rigorous evaluation of interventions in clinical research. Notably, while children and adults were studied, only two trials specifically addressed individuals aged 60 and above.

## 6. Conclusions

This systematic review suggests that non-pharmacological therapy, such as dietary intervention, may benefit patients with Gilbert’s syndrome. Such interventions should include recommendations to avoid excessive caloric restriction, mono-diets, and nutritional deficiencies (especially vitamins B12, D, and folic acid). Simultaneously, consuming biologically active compounds in vegetables and fruits (*Cruciferae*, *Apiaceous*, *Rutaceae*) could regulate serum bilirubin levels, thereby preventing jaundice episodes. It is also pertinent to emphasize to individuals with GS the importance of adopting a lifestyle that minimizes exposure to triggers of this condition. Furthermore, there remains a scientific challenge to identify food compounds capable of influencing the expression of the liver enzyme UGT, which could contribute to bilirubin regulation in individuals with GS.

## Figures and Tables

**Table 1:** Characteristics of clinical studies conducted in people with Gilbert’s syndrome regarding the impact of caloric restriction on selected metabolism parameters (n = 12).

Jadad Scale	Participants	Group	Duration	Intervention	Analysis	Results	Total Bilirubin	References
1	Adults, n = 21 Age: no data Sex: no data Ethnicity: no data Country: USA	GS (n = 7) Relatives of people with GS (n = 6) Healthy (n = 8)	2–7 days: Base-line diet 48 to 72 h Fasting diet 36 to 72 h Refeeding diet	Base-line diet 2000–3000 cal Fasting < 400 cal Refeeding 2000–3000 cal	Plasma: TB, CB	The concentration of unconjugated bilirubin (TB) in individuals with Gilbert’s syndrome significantly differed before, during, and after caloric restrictions (1.7 vs. 4.7 vs. 2.1 mg/100 mL). A relationship between calorie intake and TB levels in the blood was observed. A sudden increase in conjugated bilirubin (CB) occurred within 24 h of starting short-term fasting, followed by a significant decrease during the 12–48 h of refeeding.	TB > 1.7 mg/dL	Felsher et al. [22]
1	Adults, n = 37 Age: no data Sex: no data Ethnicity: no data Country: UK Rats Sprague-Dawley (n = 14)	Healthy (n = 12) GS (n = 10) Liver disease (n = 12) Hemolytic anemia (n = 3) Rat basal diet (n = 7) Rats starved (n = 7)	5 days	People: 2-day basal diet, reduced to 400 cal/day for 3 days indocyanine green intravenously Rats: first group basal diet, second group 3 days was starved	Venous blood: HGB, Plasma: TB, UCB, CB haptoglobin, cortisol, Hepatic UDPGT	After caloric restriction in the diet, the following changes were observed: Healthy individuals showed a significant increase in unconjugated bilirubin (UCB) concentration after 48 h. Individuals with Gilbert’s syndrome (GS) exhibited increased total bilirubin (TB) and unconjugated bilirubin (UCB) levels after 24 h. Patients with liver diseases and hemolytic anemia did not show significant changes in TB and UCB concentrations. No changes were observed in haptoglobin, cortisol levels, and the half-disappearance time of the indocyanine green injection in any of the studied patient groups. UDP-glucuronyltransferase (UDPGT) activity was significantly lower in starved rats than those with food access.	TB > 1.8 mg/dL	Owens and Sherlock [23]
2	Adults, n = 14 Age: 19–34 years Sex: 3 women; 11 men Ethnicity: no data Country: USA	Healthy (n = 5: 3 women, 2 men) GS (n = 9: men)	2 days	Diet 400 cal/day (46 g of carbohydrates, 16.5 g of protein, and 14.5 g of fat)	LDH, ALP, ALT, ASP, TB, CB, HGB, CO production Liver biopsies	In both control group patients and those with GS, significant increases in serum total bilirubin (TB) levels were observed following caloric restriction, while there were no differences in carbon monoxide (CO) production.	TB > 1.3 mg/dL	Bensinger et al. [24]
2	Adults, n = 34 Age: no data Sex: no data Ethnicity: no data Country: USA	GS (n = 11) Hemolysis and unconjugated hyperbilirubinemia (n= 10) Healthy (n = 13)	4–5 days	Dieta 2 or 3-day baseline of 2500–3000 cal/day, the caloric intake was abruptly reduced to approximately 300 cal/day for 2 days	Serum: TB, CB, Hepatic UDPG-T	Forty-eight hour fasting significantly increased TB levels in serum among individuals with Gilbert’s syndrome (from 1.5 to 3.5 mg/100 mL) and healthy individuals (from 0.5 to 0.9 mg/100 mL). Serum CB levels remained unchanged in all three patient groups. Reduced hepatic UDPG-T activity to levels observed in GS patients was observed only in two patients with hemolysis.	TB > 1.5 mg/dL	Felsher and Carpio [25]
2	Adults, n = 4 Age: 21–46 years Sex: no data Ethnicity: no data Country: USA	GS (n = 4)	About 2 weeks	Base-line balanced diet for 2 days Tested diet: low carbohydrate; low protein; low fat; isocaloric high fat, low carbohydrate, low protein; isocaloric low carbohydrate diet and a low protein	Anthropometric measurements Plasma: TB, CB	Higher TB levels were observed in individuals with GS after a low-calorie diet compared to a balanced diet. TB levels in individuals on various isocaloric diets with reduced macronutrient content were, on average, 1.1 mg/dL lower than after a low-calorie diet. A low-protein and low-carbohydrate isocaloric diet did not increase TB levels in the blood. None of the diets affected CB levels in the blood. Changes in body weight were observed only after the low-calorie diet.	TB > 1.3 mg/dL	Felsher [26]
2	Youth, adults, n = 12 Age: 14–46 years Sex: 3 women; 9 men Ethnicity: no data Country: Sweden	GS (n = 12)	3 days	Reduced caloric intake (1.7 MJ/24 h) fluid meal (50 g of fat)	Serum: bile acids, UCB	The increase in UCB concentration after caloric restriction was 140%. In fasting, before and after calorie reduction, patients had normal serum bile acid levels. Postprandial concentrations of bile acids in serum were within the normal range before and after calorie restriction.	TB > 1.5 mg/dL	Briheim et al. [27]
3	Adults, n = 28 Age: 19–37 years Sex: 5 women; 25 men Ethnicity: no data Country: Italy	GS (n = 18: 15 men, 3 women) Control (n = 10: 8 men; n = 2 women)	Of at least 2 weeks	Caloric restriction 400 cal: Diet A: 100 g of sucrose/24 h Diet B: 250 g of bran (and 100–250 cm^3^ of barium sulfate solution	Plasma: TB	In the control group, there were no differences in TB concentration after diets A and B. TB concentration significantly increased under diet A compared to diet B in individuals with GS. TB concentration did not differ significantly between groups depending on the diet applied.	TB > 1 mg/dL	Ricci and Ricci [28]
3	Adolescents/Adults n = 60 Age: 16–48 years Sex: 27 women; 33 men Ethnicity: no data Country: Italy	GS1 (n = 10 men, n = 10 women) with high TB GS2 (n = 13 men, n = 7 women) with normal TB. Control (n= 10 men, n = 10 women)	The 2 tests were performed with an interval of at least 2 weeks.	Test with nicotinic acids (NA) Test fasting/hyperbilirubinemia (FH) patients 3 days after a control normocaloric diet were given a restricted diet (400 cal/48 h)	Blood: UCB and CB	In the NA test, significant differences were observed between individuals with GS and controls in UCB, maximal increment in AUC, and retention at 240 min (excluding sex). Following calorie restriction, TB levels were higher in GS1 compared to GS2 and the control group. Serum bilirubin increased after 24 h of caloric restriction in GS1, GS2, and normal subjects but not after 48 h. Bilirubin concentrations after 24 and 48 h were higher in males with GS compared to females. The NA test was more accurate in diagnosing GS in both males and females.	TB > 1.35 mg/dL	Gentile et al. [29]
1	Adults, n = 10 Age: no data Sex: no data Ethnicity: no data Country: Turkey	GS (n = 10)	30 days	Compulsory 16 h fasting daily from 4:00 to 20:00 during Ramadan and consuming 2500 calories (30 g carbohydrates, 20 g protein, and 10 g fat)	Serum: TB, CB, UCB	After the first day of fasting, the concentrations of TB and UCB were significantly higher than before, while CB remained similar. In the following days of fasting (7, 14, 30), TB and UCB concentrations decreased to pre-fasting levels, while CB remained consistent throughout the fasting period.	TB > 1.4 mg/dL	Kapıcıoğlu et al. [30]
3	Adults, n = 146 Age: 20.7 years Sex: 146 women Ethnicity: White Country: Portuguese	Bilirubin concentration (μmmol/L) I group ≤6 (n = 49) II group 6–9 (n = 49) III group ≥ 9 (n = 48)	No data	11 h fasting	Venous blood: hematological data, Plasma: bilirubin, Buffy coat-DNA extraction, screening of TA duplication in the UGT1A1 gene, BIA, International Physical Activity Questionnaire.	Women in the second and third tertiles exhibited higher Hb, Ht, MCH, MCHC concentrations and lower platelet counts than those in the first tertile. In the third tertile, RCB was the highest. There were no differences in total and differential white blood cell counts and non-genetic factors (physical activity, smoking, oral contraception, body fat content, alcohol consumption, and fasting time). The lowest BMI was observed in the third tertile. The genetic allele c.-41_-40dupTA, Hb, BMI, and fasting time were identified as the main factors associated with bilirubin concentration in the blood.	TB > 1 mg/dL	Rodrigues et al. [31]
4	Adults, n = 120 Age 20–80 years Sex: 40 women, 80 men Ethnicity: no data Country: Austria	Group < 35 years (n = 66: GS and Control: 9 women, 24 men each) Group ≥ 35 years (n = 54: GS and Control 11 women, 16 men each)	No data	16 h fasting with a diet of 400 cal	Anthropometric measurements, BIA, questionnaire food consumption, Biomarkers of energy, carbohydrate, lipids metabolism	There were no differences between the GS and control groups in the consumption of healthy foods, snacks, red meat, alcohol, physical activity, liver enzymes, iron levels, and AMPKα1 gene expression. Individuals with GS had lower BMI, blood glucose, insulin, C-peptide, and TG levels and were less susceptible to metabolic diseases. In GS individuals, levels of p-AMPK α1/α2, -PPAR α/γ, and PgC1α were significantly higher, indicating an enhanced metabolic response to fasting.	TB > 1.0 mg/dL	Mölzer et al. [32]
2	Children, young, n = 46; Age: no data Sex: 25 girls, 21 boys Ethnicity: no data Country: Italy	Group OLT (n = 46) Control group (n = 20) non-GS OLT	1 year	1 day reduced caloric intake prolonged (14–16 h) fasting	Venous blood: hematologic parameters Serum: TB, Cb, UCB IXa, monoglucuronide, sex hormones, liver dysfunction, 2-bp (TA) insertion in the promoter of the gene	Four patients with OLT had hyperbilirubinemia, but CB levels were normal. Tests involving caloric restriction and fasting showed an increase in bilirubin levels. High relative amounts of UCB IXa and a predominance of monoglucuronide over diglucuronide were observed. Hematologic parameters and hormone levels in the blood were normal. Adult liver donors had gene mutations related to the 2-bp (TA) insertion.	TB > 1.3 mg/dL	Vajro et al. [33]

Jadad scale—0–5. Explanations are in the Abbreviations section.

**Table 2:** Characteristics of clinical trials conducted in people with GS regarding the impact of various diet variants, fruit, vegetable, and alcohol consumption on selected metabolic parameters (n = 7).

Jadad Scale	Participants	Group	Duration	Intervention	Analysis	Results	Total Bilirubin	References
4	Young adults, n = 29 Age:16–43 years Sex: 8 women, 21 men Ethnicity: no data Country: UK		About 10 days: 2 days normal diet Tested diet 1–2 day basal diet	Normal diet Tested diet: Low-energy, standard Low-energy, standard + IV glucose Low-energy, standard + IV lipid High-carbohydrate, low-lipid (fluid) High-carbohydrate, reduced-lipid Low-energy, high-lipid	Plasma: TB	The highest increase in TB concentration was observed in patients following Diet II (low-energy standard) and Diet III (low-energy, standard + IV glucose). The lowest increase in TB concentration occurred with Diet VI (high-carbohydrate, reduced-lipid). The level of hyperbilirubinemia achieved after oral carbohydrate intake was significantly lower than that observed after intravenous intake or Diet II (low-energy, standard). Diet VII (low-energy, high-lipid) significantly reduced hyperbilirubinemia to a level lower than Diet II-induced (low-energy, 400 cal).	TB > 1.7 mg/dL	Gollan et al. [34]
3	Adults, n = 191 Age: 20–40 years Sex: 112 women, 79 men Ethnicity: no data Country: USA	UGT1A1 genotype: Group 6/6 and 6/7 (n = 169) Group 7/7 (n = 22)		Assessment of the consumption of 6 groups of botanical vegetables and fruits	Demographic survey, health history, 3-D food record Indices: BMI Venous blood: TB, CB, UGT1A1 promoter genotypes.	Men had higher serum levels of TB, CB, and UCB than women. An inverse relationship was demonstrated between all bilirubin levels and the interaction of the UGT1A1*28 genotype with the intake of the botanical group *Cruciferae*. Individuals with the 7/7 genotype had reduced blood bilirubin levels with increased consumption of cruciferous vegetables.	TB > 1.6 mg/dL	Peterson et al. [35]
4	Adults, n = 70 Age: 20–40 years Sex: 35 women, 35 men Ethnicity: Caucasian, Asian, Other Country: USA	groups of genotypes UGT1A1*1/*1 (n = 29) UGT1A1*1/*28 (n = 36) UGT1A1*28/*28 (n = 5) (1) GSTM1+/GSTT1+ (n = 26) (2) GSTM1-null/GSTT1+ (n = 30) (3) GSTM1-null/GSTT1-null) (n = 14)	Each of the four diet periods lasted 14 days, with a 21-day washout period between diet periods	(1) Basal diet without fruit and vegetables; (2) Single-dose cruciferous diet; (3) Double-dose cruciferous diet; (4) Single-dose cruciferous plus apiaceous vegetable diet	*GSTM1, GSTT1* genotypes UGT genotyping 3-d food record Plasma: TB, CB Urine: total isothiocyanates Indices: BMI	The decrease in serum bilirubin concentration in response to all three diets compared to the basal diet was particularly pronounced in individuals with the UGT1A1*28/*28 genotype and those consuming a double dose of cruciferous plants, which enhances UGT1A1 activity. Although UGT1A1 activity, measured by serum bilirubin, was higher in individuals with the GSTM1-null genotype than those with GSTM1+, this response was not statistically significant.	TB > 1.2 mg/dL	Navarro et al. [36]
3	Adults, n = 239 Age: 20–40 years Sex: 147 women, 146 men Ethnicity: Caucasian, Asian, Other Country: USA	UGT1A1 genotype: Group 6/6 Group 6/7 Group 7/7	No data	Week	Food Frequency Questionnaire 6 botanical families 3-D food record; anthropometric measurements Plasma: TB, UGT genotyping	Gender (men), race (Asian), and the UGT1A1 genotype were associated with serum concentrations of TB, CB, and UCB. There was no association between the consumption of vegetables and fruits (assessed by FFQ) and TB levels. In women, an interaction was found between the UGT1A1 (7/7) genotype and the consumption of citrus fruits (assessed by FFQ) and *Rutaceae* (assessed by 3DFR), which was related to TB and UCB levels.	TB > 1.0 mg/dL	Saracino et al. [37]
3	Children, n = 29 Age: 8–17 years; Sex: 10 girls, 19 boys Ethnicity: no data Country: Serbia	GS (n = 29)	9 months	24 h of fasting and after 24 h of hypercaloric intake	Questionnaire related to urinary tract Blood: glucose, TB, CB Urine: ketone bodies uroflowmetric test	There was a normal uroflowmetric pattern in 69% of children and after 24 h of hypercaloric diet in all children. Children with an abnormal uroflowmetric pattern had higher CB concentrations after 24 h of fasting than children with a normal pattern. There were no differences in TB and UCB concentrations. In children with normal and abnormal UFC, pre-fasting CB concentration was lower than post-fasting, and glucose concentration was higher. VC in children was normal after 24 after 24 h fasting and after 24 h of hypercaloric intake.	TB > 1.3 mg/dL	Stojanović and Vukavić [38]
3	Adults, n = 30 Age: 18–71 years Sex:12 women, 18 men Ethnicity: no data Country: Germany	GS (n = 30)	No data	After 2 days of abstinence participants drank 0.1 L of sparkling wine (9 g ethanol)	Anthropometric measurements; Lifestyle and Eating Habits Questionnaire Urine: EtG, EtS, creatinine Venous blood: morphology, ethanol, TB, CB, liver enzymes	In 69% of the children, the uroflowmetric pattern was expected, and after 24 h on a hypercaloric diet, it was typical in all children. Children with an abnormal uroflowmetric pattern had higher CB levels after 24 h of fasting than children with a regular pattern. There were no differences in TB and UCB levels. CB levels were lower pre-fasting than post-fasting in children with normal and abnormal UFC, while glucose levels were higher. Bladder capacity (VC) in children was average after 24 h of fasting and after 24 h of hypercaloric intake.	TB > 0.67 mg/dL	Huppertz et al. [39]
2	Obese adults n = 10; Age: 58.4 years Sex: 7 women, 3 men; Ethnicity: no data Country: USA	GS (n = 10) with cirrhosis and nonalcoholic steatohepatitis (NASH).	>4 year (8.7 ± 1.4 year)	Bariatric surgery (laparoscopic sleeve gastrectomy or Roux-en-Y gastric bypass)	Body mass, weight; Venous blood: HbAC1, Plasma: albumin, TB, Indices: TBWL, BMI, INR, MELD-Na	The patient with GS experienced a TBWL of 23.5% and a reduction in BMI of 7.7 kg/m^2^. Despite a decrease in HbAC1 concentration of 1.3%, insulin treatment was necessary. An increase in TB concentration of 1.4 mg/dL of blood was observed. The MELDNa Score for End-Stage Liver Disease in the patient with GS was 14, indicating a 6% mortality risk.	TB > 0.6 mg/dL	Izzy et al. [40]

Jadad scale—0–5. Explanations are in the Abbreviations section.

## Data Availability

The bibliographic query in the Repository for Open Data https://doi.org/10.18150/RA5ILC.

## Abbreviations

ALP—Alkaline phosphatase; ALT—alanine transaminase; AMPK—heterotrimeric AMP—activated ser/thr kinase; AMPK1a expr.: AMPK gene expression as relative quantification; AST—aspartate aminotransferase, AST; BIA—bioelectric impedance analysis; BMI—body mass index; BW—body weight; CB—conjugated bilirubin (direct); CLDQ—Chronic Liver Disease Questionnaire; CO—carbon monoxide; CVD—cardiovascular disease; EtG—ethyl glucuronide; EtS—ethyl sulfate; FH—fasting/hyperbilirubinaemia test; GD—Gilbert’s disease; GGTP (γ-GT)—gamma-glutamyl trans peptidase; GS—Gilbert’s syndrome; GST—glutathione S-transferases; HbA1C—glycated hemoglobin; HCV—hepatitis C virus; HGB—hemoglobin; LDH—lactate dehydrogenase; NA—nicotinic acid; NAFLD—non-alcoholic fatty liver disease; NSAIDs—non-steroidal anti-inflammatory drugs; NASH—non-alcoholic steatohepatitis; OLT—orthotopic liver transplant; p-AMPK α1/α2—phosphorylated 5′-AMP activated kinase; PgC1α—peroxisome proliferator-activated receptor c coactivator 1; pPar α—phosphorylated peroxisome proliferator activated receptor alpha; pPpar γ—phosphorylated peroxisome proliferator activated receptor gamma; PRISMA—Preferred Reporting Items for Systematic Reviews and Meta-Analysis; ROS—reactive oxygen species; SF-36v2—Short form-36 Health Survey test, version 2; TA—thymidine–adenosine pairs; TB—total bilirubin; TBWL—total body weight loss; TC—total cholesterol; UCB—unconjugated bilirubin (indirect); UDPGA—uridine diphosphoglucuronic acid; UDPG-T—hepatic bilirubin uridine diphosphate glucuronyltransferase; UFV—urinary flow curve; UGT1—UDP-Glucuronosyltransferase 1; UGT1A1—uridine 5-diphosphate-glucuronosyltransferase 1A1 genotype; VC—volume capacity.
